# Alpine grassland community productivity and diversity differences influence significantly plant sexual reproduction strategies

**DOI:** 10.1093/pnasnexus/pgae297

**Published:** 2024-08-09

**Authors:** Xiaomei Kang, Yanjun Liu, Xinyang Wu, Jiachang Jiang, Lijie Duan, Aoran Zhang, Wei Qi

**Affiliations:** State Key Laboratory of Herbage Improvement and Grassland Agro-ecosystem, College of Ecology, Lanzhou University, No. 222 South Tianshui Road, Lanzhou 730000, China; State Key Laboratory of Herbage Improvement and Grassland Agro-ecosystem, College of Ecology, Lanzhou University, No. 222 South Tianshui Road, Lanzhou 730000, China; State Key Laboratory of Herbage Improvement and Grassland Agro-ecosystem, College of Ecology, Lanzhou University, No. 222 South Tianshui Road, Lanzhou 730000, China; Gansu Provincial Extension Station of Grassland Techniques, No. 92 West Railway Station Road, Lanzhou 730000, China; Gansu Provincial Extension Station of Grassland Techniques, No. 92 West Railway Station Road, Lanzhou 730000, China; State Key Laboratory of Herbage Improvement and Grassland Agro-ecosystem, College of Ecology, Lanzhou University, No. 222 South Tianshui Road, Lanzhou 730000, China; State Key Laboratory of Herbage Improvement and Grassland Agro-ecosystem, College of Ecology, Lanzhou University, No. 222 South Tianshui Road, Lanzhou 730000, China

**Keywords:** community structure, flowering phenology, pollinator competition, reproductive biomass allocation, species biologic attribute

## Abstract

Whether and how community structure variation affects plant sexual reproduction is crucial for understanding species’ local adaptation and plant community assembly, but remains unrevealed. In Qinghai-Tibetan grassland communities that differed in aboveground biomass (AGB) and species diversity, we found significant influence of AGB on both species’ reproductive biomass allocation (RBA) and flowering and fruiting time, but of species diversity only on species’ reproductive time. In high-AGB or high-diversity communities, smaller and earlier flowering species generally advanced their reproductive phenology and increased their reproductive allocation for maximizing their reproductive success, whereas larger and later flowering species delayed their reproductive phenology and decreased their reproductive allocation for maximizing their vegetative growth and resource competition. This change in reproductive allocation with the variation in community structures was more pronounced in nonclonal as compared to clonal plant species. Thus, we evidence an important influence of community structure on plant sexual reproduction strategies, and the pattern of the influence depends largely on species biological attributes.

**Significance Statement**

Plant reproductive tradeoffs, i.e. allocating time and biomass to reproduction, are one of the strongest bioindicators of environmental change. Co-existing species often compete for available resources and pollinators, thus, their reproductive tradeoff is expected to vary with community structure. In an alpine grassland, we found significant effects of community productivity on both species’ reproductive time and biomass allocation, but biodiversity effects only on species’ reproductive time. Reproductive tradeoffs difference among species with different biologic attributes generally increased along community productivity and diversity gradients. Overall, these results demonstrate that biological attribute-dependent species reproductive tradeoff differentiation and the shift of the reproductive tradeoff with community structure variation should be an important mechanism of community assembly.

## Introduction

Regarding plant reproduction, tradeoffs are ubiquitous (1, 2). An individual's size is limited by available resources, and plants often face a biomass tradeoff in how to allocate their limited biomass to different organs. If biomass allocation to reproductive (or vegetative) organs is too high, it is not conducive to individual plant growth and competition (or seed yield and quality), and ultimately, fitness (3–5). Thus, for maximizing fitness, individual plants often adopt an optimal biomass allocation pattern (1, 6). Because the suitable growing season is often limited, plants also face a time tradeoff, that is, how to allocate growing season to different successive events of the vegetative and reproductive phases (7–9). An individual plant that flowers too early, before it has time to accumulate sufficient assimilated products and materials, will have a limited seed production ability. Others that delay flowering might accumulate abundant materials for seed production, but often run out of time to use it before the end of the season (8–10). Additionally, premature fruit ripening might lead to inadequate time for seed development, but delayed fruiting usually increases the risk of plant reproductive failure and decreases seed dispersal time (3, 7, 11, 12). Thus, studies focused on plant sexual reproduction strategies related to time and biomass tradeoffs, i.e. plant reproductive phenology and biomass allocation, are crucial in understanding plant species local adaptation and evolution.

Plant sexual reproduction strategies are affected by a variety of environmental factors, including temperature, precipitation, soil fertility, and photoperiod, which are reliable signals of available resources and seasons (7, 9, 12). Comparatively, community structure should change the strategies due to its significant effects on biotic interactions and plant life-history strategies (10, 13–16), but has been considerably less investigated. Several biodiversity manipulation experiments have shown that with increase in co-occurring plant species, the decrease in flower visit frequency due to the increase of interspecific competition for pollinators and the increase of the probability of heterospecific pollen transfer generally delay most plant species’ flowering time to maintain their high seed set (15, 17, 18). Some nutrition addition or planting experiments have also revealed that increasing community productivity or individual density generally increases interspecific resource competition and thus decreases individual plant reproductive biomass allocation (RBA; 3, 5, 19). However, for natural grasslands, it remains largely understudied whether significant variation in plant community structure is sufficient to cause detectable shifts in plant reproductive phenology and biomass allocation (10).

**Fig. 1.**
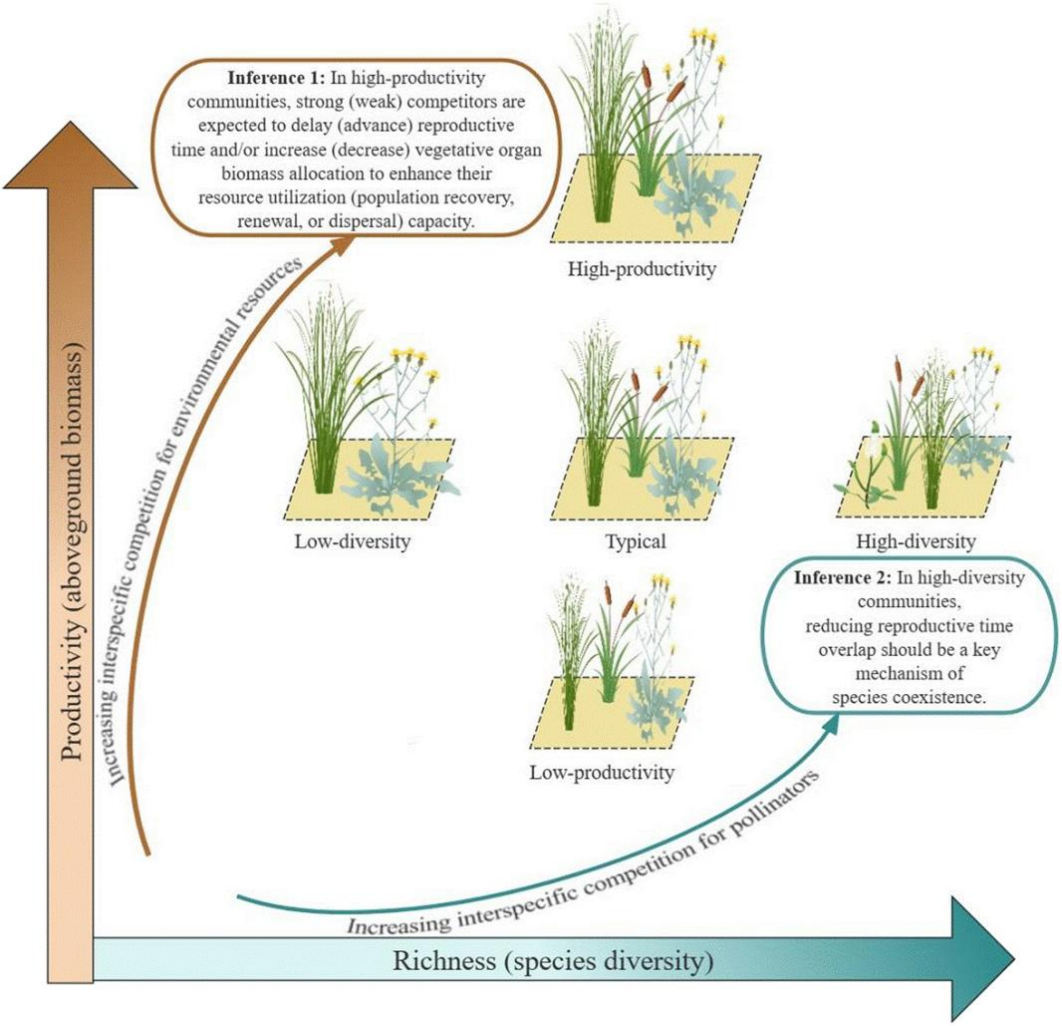
Schematic diagram of the impact of changes in productivity and diversity of alpine grassland communities on plant species reproduction strategies (assuming that all grassland communities have similar climatic conditions and soil properties).

In this study, we selected five adjacent Qinghai-Tibet alpine grassland communities with similarity in climate conditions, topographic conditions, soil properties, and grazing intensity but difference only in structures (i.e. a typical with middle productivity and diversity, high-productivity, low-productivity, high-diversity, and low-diversity community). For all communities, we measured RBA and peak flowering/fruiting time for 26 common species. Theoretically, with increasing community productivity, vegetation height, canopy density, and the size of the root system generally increase, so interspecific environmental resource (e.g. light and soil nutrition) competition should also increase. An increase in species diversity, however, should enhance interspecific competition for pollinators in the case that stressful alpine environments, low temperature and short growing season length tend to decrease or limit flowering duration length and animal pollinator number and activity (3, 15, 17, 18, 20, 21; Fig. 1). Thus, we hypothesize 1 that changes in community productivity or species diversity can affect plant species reproductive phenology and biomass allocation (H1). Furthermore, since species biological attributes, especially the attributes related to plant individual development and reproductive success, are often closely related to their flowering and fruiting time and other reproduction strategies (4, 12, 22, 23), we hypothesize 2 that these attributes will affect the response of plant reproductive phenology and biomass allocation to the changes in plant community structures (H2).

## Results

**Fig. 2.**
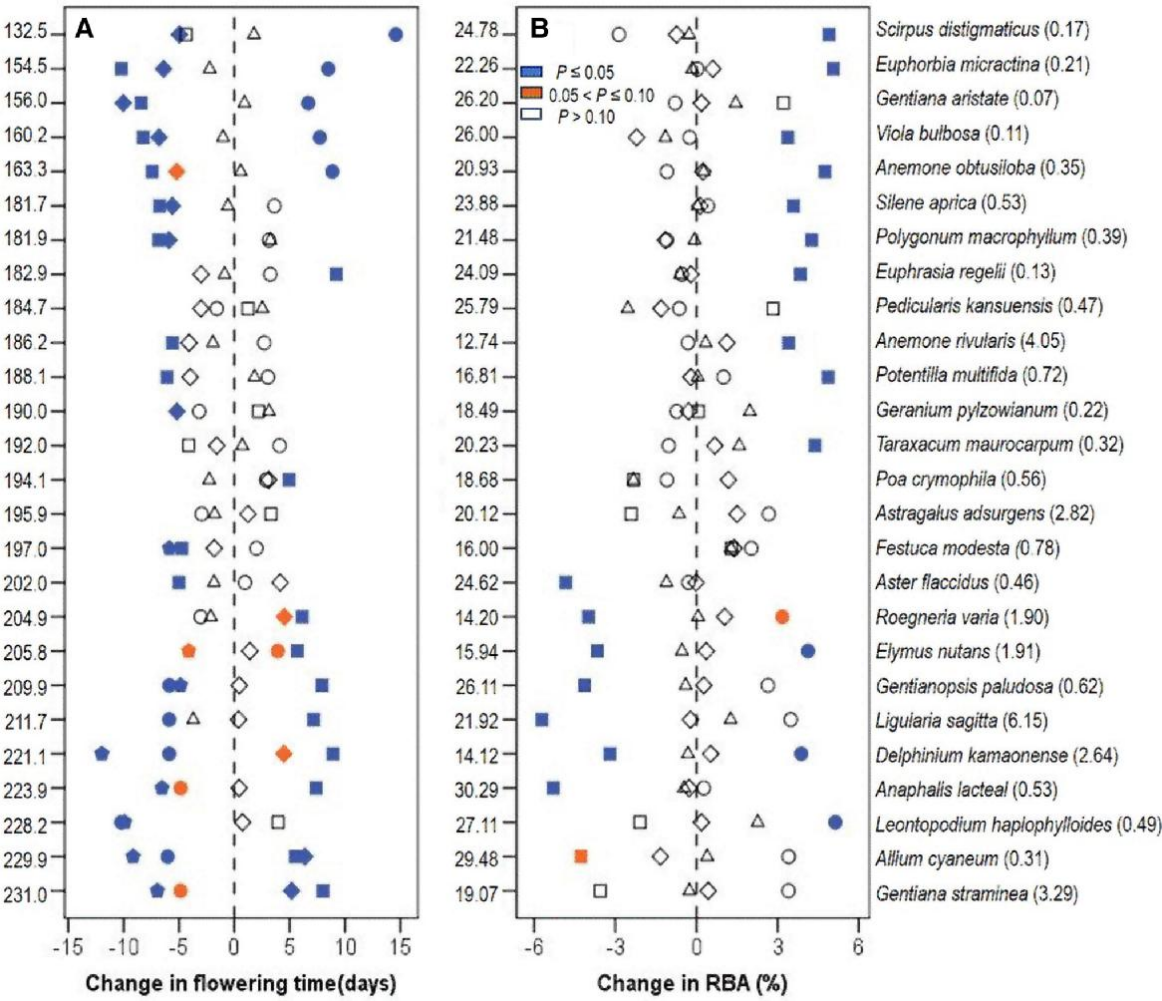
Change in peak flowering time A) and RBA B) of 26 grassland species in the high-productivity (squares), low-productivity (circles), high-diversity (diamonds) or low-diversity (triangles) community relative to the typical community (control). A positive (or negative) change indicates that variation in community productivity or diversity delays (or advances) flowering time or increases (or decreases) RBA. Species are presented on the right side of the figure in order of their mean individual flowering time (Julian day) in the control. Numbers on the left side of each figure (A, B) represent the mean individual flowering time (A) and RBA (%, B) of each species, respectively, in the control. Mean individual plant size of each species in the control is represented on the rightmost side of figure after the species name.

The effect of community structure on plant sexual reproduction strategies (RBA and flowering or fruiting time) was significantly different among species. Early (or late) flowering species tended to advance (or delay) flowering and fruiting time, but increase (or decrease) RBA in response to high community productivity (Figs. 2 and S5). Response of the above strategies of these species to low community productivity was usually opposite to that to high community productivity, except for the overall nonsignificant RBA response for early flowering species and fruiting time response for late flowering species. Species richness rarely affected species’ RBA. In contrast, high species richness advanced (or delayed) early (or late) flowering species’ flowering and fruiting, but low species richness advanced late flowering species’ flowering and fruiting (Figs. 2 and S5).

**Fig. 3.**
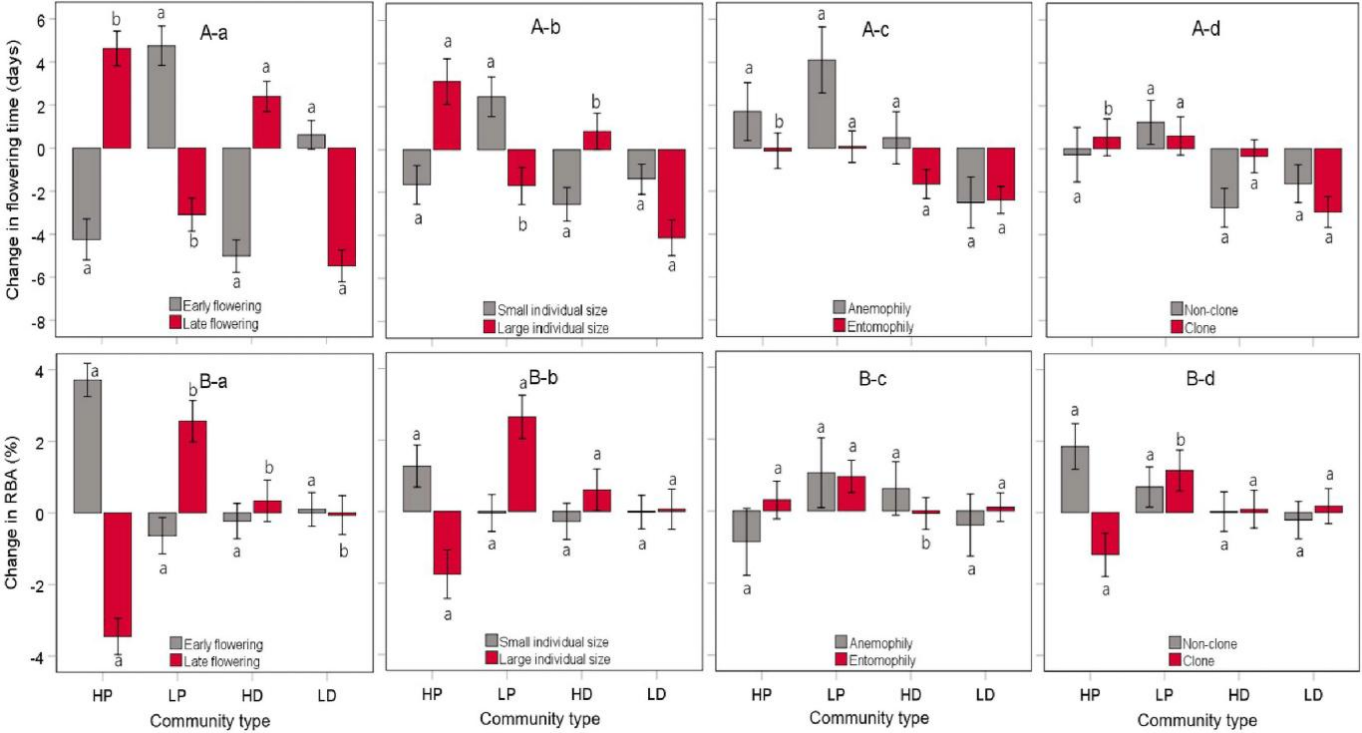
Change in peak flowering time A) and RBA (B) for species with different biological attributes (a, early or late flowering; b, small or large individual size; c, anemophily or entomophily; d, clone or nonclone) in the high-productivity (HP), low-productivity (LP), high-diversity (HD), or low-diversity (LD) community relative to the control. Error bars show the 95% confidence intervals (CIs). Different letters indicate significant difference between species with different biological attributes in their flowering time or RBA change with community productivity or diversity.

The response of plant species reproductive phenology and biomass allocation to community structure also depended on individual plant size. In the high-productivity community, small-size species significantly increased their RBA, whereas in the high- and low-productivity community, large-size ones decreased and increased their RBA, respectively (Fig. 3). Large-size species delayed (or advanced) flowering in the high (or low) productivity or diversity community, whereas small-size ones advanced flowering in the high-productivity, high-diversity, or low-diversity community but delayed flowering in the low-productivity community. Large- and small-size species delayed and advanced fruiting in the high-productivity or diversity community, respectively, whereas they both delayed fruiting in the low-productivity community but advanced fruiting in the low-diversity community. Anemophilous and entomophilous species had similar RBA responses to variation in community structure, but anemopholius species shifted (advanced) flowering and fruiting to a greater extent than entomophilous ones with increasing community diversity and productivity, and decreasing community productivity (Fig. 3). Compared with clonal species, nonclonal species significantly increased RBA in the high-productivity community and advanced flowering and fruiting in the high-diversity community (Fig. 3).

## Discussion

Because selected grasslands had similar climate, topographic and soil conditions and grazing intensity (seen in SI Appendix 1), the significant difference among communities in RBA and flowering/fruiting time supported our first hypothesis of the existence of community factors in plant reproductive strategies. Productivity affected both species’ RBA and reproductive time, whereas species diversity mainly affected species’ reproductive time. Variation in grassland productivity and species richness often changes the pattern or degree of interspecific competition for available environmental resources and pollination vectors (3, 14, 15, 17, 18, 20), respectively. These findings imply that grassland species tend to vary their reproductive strategies related to both biomass and time tradeoffs to adapt to interspecific environmental resource competition, but to shift their reproductive time to decrease or avoid potential competition with co-existing species for the same pollinators, being consistent with our two inferences in Fig. 1.

Overall, our results also support the second hypothesis because species biotic attributes significantly affected the response of their reproductive phenology and biomass allocation to community structure variation, except for the nonsignificant response of their RBA to species diversity variation. The exception implies that, without community productivity variation, the amount of resources allocated to the reproductive organs of a plant individual do not change due to species biological properties and the composition and number of their co-existing species. Among all species, early flowering species usually flowered and fruited earlier, while late flowering species flowered and fruited later to cope with increasing community productivity and species diversity. For the cold alpine grassland species, early flowering means short-time vegetative growth and limited dry matter accumulation for flower development which leads to short stature and small or few flowers (8, 9). Therefore, species are at a competitive disadvantage for animal pollinators or environmental resources when they co-exist with other species. In this case, with increasing community biomass and species diversity, earlier flowering for early flowering species can improve their pollination success and resource acquisition before other species grow tall, whereas earlier fruiting can prevent reproductive failure caused by lack of access to (or low competitiveness for) sufficient environmental resources when or after other species grow up. Thus, an earlier reproductive phenology for early flowering species in high-productivity or diversity communities may follow a strategy similar to “fugitive-coexistence” that inferior competitors (plant species) prevent being eliminated by superior ones by decreasing/escaping direct interspecific adult competition in time and space and (or) increasing the survival or colonization ability of offspring (24–27). This strategy may also explain why early flowering plants in the higher productivity communities allocate more biomass to reproduction (e.g. seed development; 26). Additionally, this results also indicates an increase in flowering or fruiting time difference among early and late flowering species with increasing species diversity, which implies that reducing reproductive time niche overlap (or increasing reproductive time niche differentiation) may be an essential prerequisite for multi species co-existence (14, 15, 20, 21, 24, 28; Fig. 1).

In most plant communities, small-size species are generally typical inferior competitors and r-strategists who should invest more time and biomass into reproduction with increasing competition or other stresses (22, 29, 30). This was confirmed by our results that small-size species advance their flowering and increase RBA and reproductive time (i.e. the time length between flowering and fruiting) in response to increasing community productivity. Large-size species, however, show the features of K-strategists that delay reproduction and decrease RBA in high-productivity communities and delay reproduction in high-diversity communities to maximize their individual competition ability (2, 3, 5, 19, 29; Fig. 1). Additionally, the shift (delay) of flowering and fruiting time of large-size species with increasing species diversity was more significant than that of small-size ones, suggesting that large-size species have higher plasticity in reproductive time to avoid high pollinator competition or invalid pollination (e.g. pollen being transferred to the stigma of heterogeneous individuals; 17, 18, 21, 31, 32).

We also considered the effect of species’ pollination type and clonality on the response of their reproductive strategy to community structure variation. Theoretically, because entomophilous species reproductive structure or flowering phenology needs to coevolve or covary with animal pollinator morphology and activity rhythms (10, 13, 20, 24, 32, 33), their reproductive strategy variation with community productivity or diversity gradients is expected to be lower than anemophilous species. This expectation was supported by most of our results except for significantly high species flowering time variation of entomophilous species with increasing community species diversity. This implies that high interspecific competition among entomophilous species for animal pollinators increases their flowering time shift to decrease the probability of heterospecific pollen transfer and increase reproductive success (10, 20, 21, 31, 32). In addition, nonclonal species differed from clonal ones in the significant increase in their RBA with increasing community productivity and significant advance in their flowering and fruiting time with increasing community diversity. The difference may be because nonclonal species’ population regeneration depends only on sexual reproduction and seed production. In this case, strategies to increase seed production in high competition conditions, i.e. increasing RBA in high resource competition communities or extending pollination time (e.g. advancing flowering) in high pollinator competition communities, may be more important for population maintenance or regeneration in nonclonal, than in clonal species.

In conclusion, species’ individual size and flowering time are the main factors affecting the response of their reproductive strategy to community structure variation. Overall, with increasing community productivity and species diversity, all species can be divided into two categories: small-size and early flowering species maximizing reproductive success and large-size and late flowering species maximizing vegetative competition. The study demonstrates that the biological attribute-dependent species reproductive strategy differentiation and shift in reproductive strategy with community structure variation should be an important community assembly mechanism, but it is often easy to be ignored.

## Materials and methods

### Field site, community investigation, and soil sampling

Our study was conducted in 2021 at an alpine grassland site (34°48′ 57″N, 103°02′32″E, 3060–3190 m a.s.l) on the east edge of Qinghai-Tibet Plateau, China. The climate is cold and semi-humid and the grassland type is alpine meadow (details in SI Appendix 1). Over c. 350 ha, we established five 10 m × 10 m plots at 300–2,000 m apart, representing different community structures. These plots were located in the relatively open areas with no significant slope and aspect; in winter, they were grazed at similar and low intensity. These plots were: typical (control) with mid diversity (20.50 ± 1.00 species per 0.25 m^2^, same hereinafter) and productivity (112.92 ± 5.19 g), high-productivity (141.85 ± 6.64 g), low-productivity (90.51 ± 7.06 g), high-diversity (24.95 ± 1.33 species) and low-diversity (17.40 ± 0.97 species) community, respectively. The high- and low-diversity (or high- and low-productivity) communities had similar productivity (or diversity) to the control. In each plot in August, twenty 0.5 m × 0.5 m quadrats were established and investigated. The details on study site, methods of community investigation and soil sampling and measurement, and the results of the comparison among plots in community structure and soil properties were seen in SI Appendix 1. The results showed similar soil properties among plots.

### Plant phenology and reproductive allocation measurement

In the control, high-productivity, low-productivity, high-diversity, and low-diversity grassland plots, we found 45, 52, 37, 43, and 44 angiosperm species, respectively. Among these species, 26 were common to all plots. Each common species in each plot was named as a population (altogether 130 populations). For each population, 26–30 individuals (altogether 3,672 individuals) outside the quadrats but within the plot and its 2 m extension were selected and aspects of their reproductive phenology recorded approximately weekly for total 25 times from the end of April to the middle of October. In each census, plant species or individuals would not be observed until they approached the peak flowering or fruiting time. The peak of an individuals’ flowering or fruiting was defined as the date (Julian day) when it reached its maximal number or proportion of open flowers and ripe fruits, respectively (14, 15). Specifically, for a plant individual, if nearly or more than half of its flowers were open in a certain census, Julian day of this census was defined as its peak flowering time and it was excluded from subsequent census until its peak fruiting time (nearly or more than half of its fruits were ripe). Population-level peak flowering or fruiting time was calculated from the individual mean. Because parts of individuals failed to reproduce, 2,993 and 2,667 individuals were finally recorded for peak flowering and peak fruiting, respectively. For each population, 16–23 individuals (altogether 2,518 individuals) at fruit ripening stage were randomly selected to measure biomass allocation. In the field, each individual was harvested by cutting the stem at the soil surface and then divided into three parts: fruits, leaves, and others (main stem). Each plant sample part was weighed after drying for 48 h at 60°C. Taking the mean flowering time (Julian day = 192.66), and mean individual plant size (log value = −0.224 g) of all common species as the boundary, we divided these species into early or late flowering, and small or large individual size species, respectively. This time boundary is extremely close to the median of local grassland plant growth season (around from Julian day of 95 to 290). At this time, the air temperature is usually the highest and the plant growth rate fastest of the year. Thus, species flowering before this time may be a “reproduction-priority” species, whereas species flowering after this time may be a “growth-priority” species. This individual plant size boundary can roughly divide all species into two groups, large ones mainly utilizing resources (e.g. light or pollinators) in the upper layers of the community and small ones mainly utilizing resources in the middle and lower layers. Besides, all common species were divided these species into anemophilous and entomophilous species and clonal and nonclonal species according to their pollination type and clonality, respectively. Details on the methods of plant phenological observation and RBA measurement are in SI Appendix 1.

### Data analyses

For each common species, a one-way ANOVA with Tukey's HSD test were used to analyze the effect of community structure on individual RBA and flowering and fruiting time. We then calculated the difference (change) in each species reproductive trait values between the high-productivity, low-productivity, high-diversity, or low-diversity communities and the control (typical community). A positive (or negative) difference indicates that variations in community productivity or diversity delay (or advance) flowering/fruiting time or increase (or decrease) RBA. We performed another one-way ANOVA to examine whether species biological attributes (early or late flowering, small or large individual size, anemophily or entomophily, clone or nonclone) significantly affected the difference.

## Supplementary Material

pgae297_Supplementary_Data

## Data Availability

All data generated or analyzed during this study are included in Supplementary material.
