# Supplementary material for: Alpine grassland community productivity and diversity differences influence significantly plant sexual reproduction strategies
Source: PNAS Nexus. 2024 Aug 9;3(8):pgae297. doi: 10.1093/pnasnexus/pgae297 (PMC11310588; doi:10.1093/pnasnexus/pgae297)

**
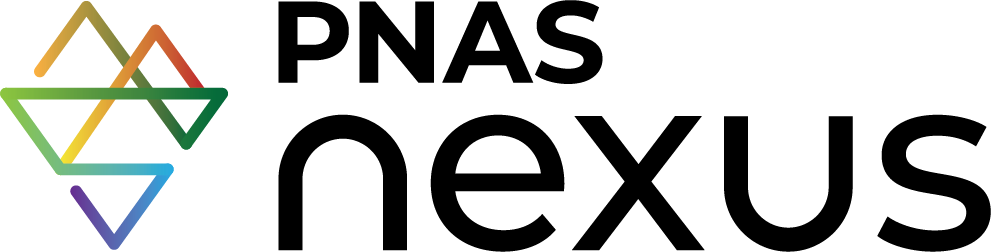
**

**Supplementary Information for**

Alpine grassland community productivity and diversity differences influence significantly plant sexual reproduction strategies

Xiaomei Kang1, †; Yanjun Liu1, †; Xinyang Wu1; Jiachang Jiang2; Lijie Duan2; Aoran Zhang1; Wei Qi1*

† Xiaomei Kang and Yanjun Liu contributed equally to this paper

***Corresponding author:** Wei Qi

**Email:** qiw@lzu.edu.cn

**This PDF file includes:**

Supplementary text

Figures S1 to S2

SI References

**Supplementary Information Text**

Appendix 1

**The details on study site, community investigation and soil sampling and the comparison among plots in community structure and soil properties**

**Study site**

The study site was conducted in 2021 at an alpine grassland site (34°48′ 57″N, 103°02′32″E, 3060-3190 m a.s.l) on the east Qinghai Tibet Plateau, China. The climate is continental with cool and semi-humid characteristics, with a mean annual temperature (MAT) of 1.1 °C, mean temperature of the warmest quarter (MTWQ) of 9.2 °C and the coldest quarter (MTCQ) of - 7.1 °C, mean annual precipitation (MAP) of 577 mm, precipitation of the wettest quarter (PWQ) of 314 mm and driest quarter (PDQ) of 19 mm, mean annual sunshine hours (MASH) of 2387, and mean relative humidity of 54.6%. The sites covered about 350 ha, and located in the relatively open areas with no significant slope and aspect; in cold seasons (from October 20 to March 30), pastures were grazed by yaks at similar and low grazing intensity (c.a. 1.3-1.6 yaks per ha). Overall, studied grassland is alpine meadow. Due to the proximity of the study site to the subalpine region, mountain meadow species and alpine meadow species coexist in a community, resulting in a high species diversity and the lack of obivously dominant species. The main plant genera of studied grassland were *Elymus, Festuca, Gentiana, Poa, Anemone, Polygonum and Potentilla*.

**Plot selection, community investigation and soil sampling**

In the study, we needed to select five 10 m × 10 m plots representing different community structures with an interval of 300-2000 m: a typical (control), high-diversity, low-diversity, high-productivity and low-productivity community, respectively (Fig. S1). The high- (or low-) diversity community should have significant higher (or lower) species richness (species number per 0.25 m^2^) than, but similar aboveground biomass (AGB, g/0.25 m^2^) with, the typical community; whereas the high- (or low-) productivity community should have significant higher (or lower) AGB than, but similar species richness with, the typical community. These plots should have similar soil properties to avoid the possibility that among-community difference in species reproductive traits or strategies was cause by available soil nutrition. In order to meet the above criteria, plots were selected in 2020 (the year before the field study). Specifically, we first selected a plot with middle species richness and middle AGB as a typical grassland (control). Then, for each of other types of community (high-productivity, low-productivity, high-diversity and low-diversity community), four plots were preselected. For each of all 17 selected or preselected plots, species richness and AGB of eight 0.5 m × 0.5 m quadrats were set and investigated on August 11-24. After community investigation, soil was sampled and measured in each quadrat (method of soil sampling and measurement were same as below). We compared community structure and soil properties among plots, and found that one high-productivity, two low-productivity, two high-diversity and one low-diversity plots met our selection criteria. Among these two low-productivity (or high-diversity) plots, the plot with lower AGB (or higher species richness) was finally selected.


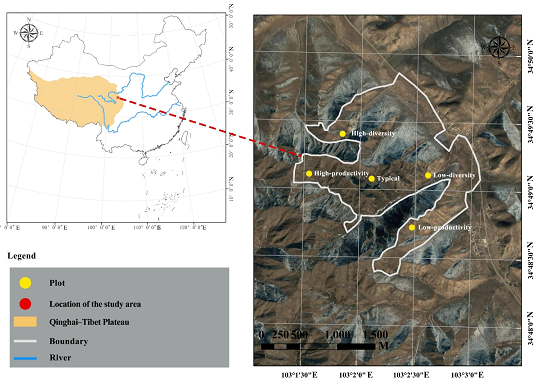

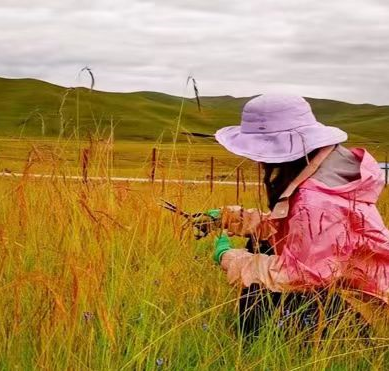

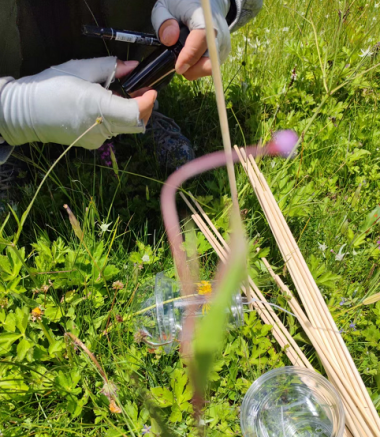

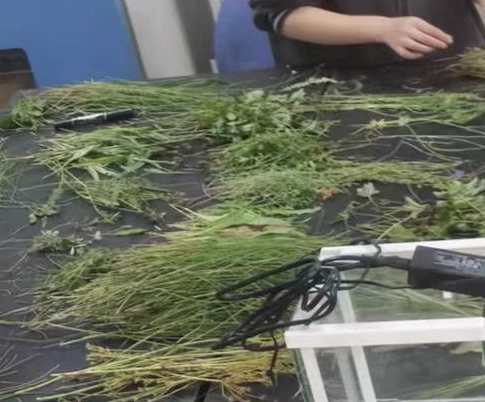

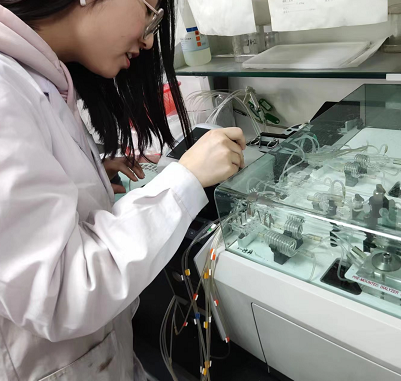


Fig. S1. Location of the studied site and photograph of field and indoor work.

On April 18-22 of 2021, each of five selected (in 2020) 10 m × 10 m plots was divided into twenty 2.5 m × 2 m subplots. At the center of each subplot, one 0.5 m × 0.5 m quadrat was set. During August 9-30 of 2021, we recorded the total canopy cover, species number, the percent cover and aboveground biomass of each species in each quadrat. Aboveground biomass was clipped to the soil level, weighed after removing any dead parts, and dried at 75 °C for 48 hours to a constant weight. We also considered the Shannon-Weiner and Pielou evenness index of each quadrat. They are calculated as: Shannon = - ∑ (pi× ln pi) and Pielou = Shannon/ln S, where pi is biomass of species i and S is the number of species (i.e., richness) in the community. After community investigation, soil was sampled in each quadrat with three depths (0-10-20-40 cm). We applied the potentiometric method (water: soil ratio at 2.5:1) to test soil pH. We used an elemental analyzer (Vario EI, Elementar, Germany) to measure soil total carbon (STC, %) and nitrogen (STN, %), and ICP-MS (Spectro Arcos EOP) to measure soil total phosphorus (STP, mg/g; data of community structure and soil properties of all quadrats were seen in Supplementary Data 1).

**Results**

**The comparison among plots in community structure**

Consistent with our plot selection criterion, the high-diversity (24.95±1.33 species and 118.67±6.51 g per 0.25 m^2^, same hereinafter) and low-diversity (17.40±0.97 species and 108.91±5.32 g) communities separately had significantly higher and lower species richness than, but similar AGB with, the typical community (20.50±1.00 species and 112.92±5.19 g); whereas the high-productivity (21.10±1.04 species and 141.85±6.64 g) and low-productivity (20.05±1.08 species and 90.51±7.06 g) community separately had significantly higher and lower AGB than, but similar species richness with, the typical community (Fig. S2-A, B). In addition, no significant difference was found among five communities in Shannon-Weiner index (Fig. S2-C), but the Pielou evenness index was highest in low-productivity and high-diversity communities, then in typical and low-diversity ones, and lowest in high-productivity ones.


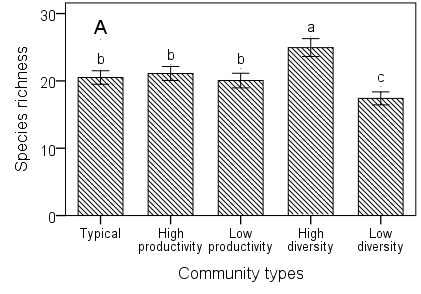


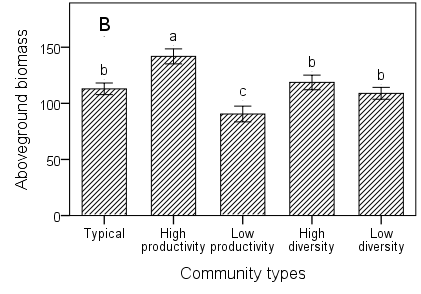


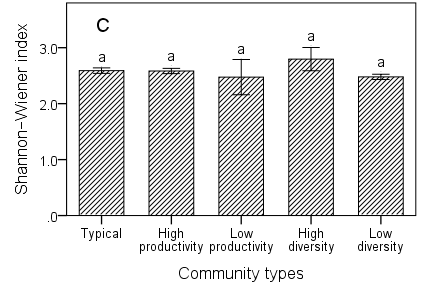


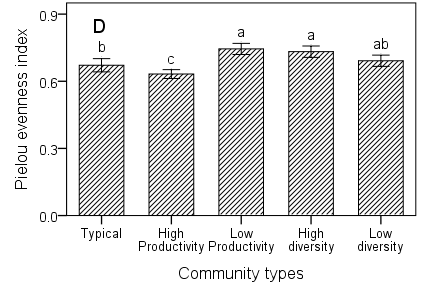


Fig. S2. The comparison (mean ± 95%) among five community types in their species richness (A; species/0.25 m^2^), aboveground biomass (B; g/0.25 m^2^) Shannon-Weiner index (C) and Pielou evenness index (D). The different lowercase letters indicated signiﬁcant difference in diversity indices among five grassland communities (plots).

**The comparison among plots in soil properties**

The difference in soil pH (Fig. S3-A), STC (Fig. S3-B), STN (Fig. S3-C) and STP (Fig. S3-D) at three depths of soil layer was generally non-significant among five types of grassland community except for a higher STC and STN in high-productivity grassland at 20-40 cm soil layer. The results indicated overall similar soil properties for five types of grassland community.


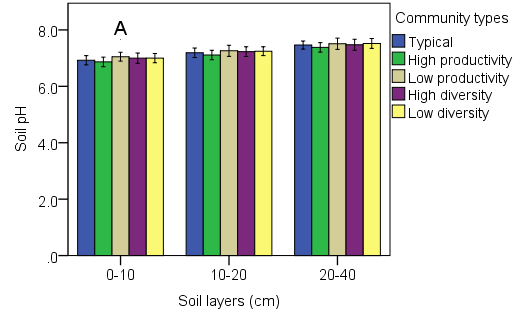


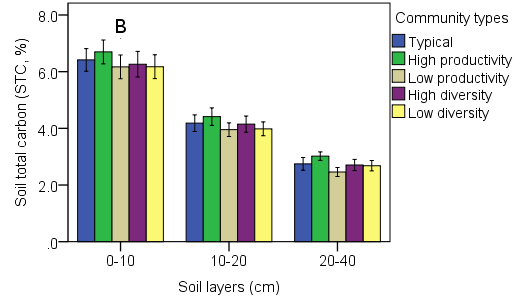


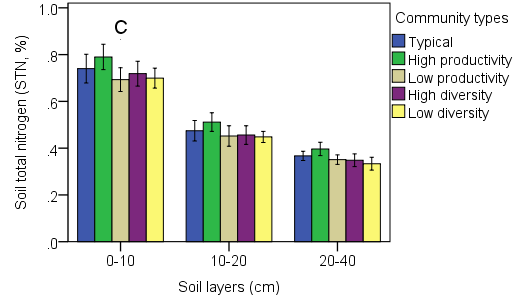

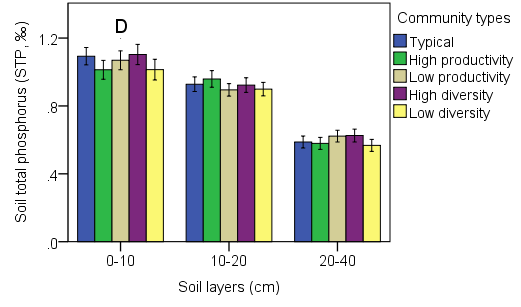


Fig. S3. The comparison (mean ± 95%) among five community types in their soil pH (A), STC (B), STN (C) and STP (D)

Appendix 2

**The methods of plant phenological observation and reproductive biomass allocation measurement**

**Plant reproductive phenology observation**

According to community investigation in 2020 and 2021, there were 45, 52, 37, 43 and 44 angiosperm species in the typical, high-diversity, low-diversity, high-productivity and low-productivity grassland plot, respectively. Among these species, 26 were common in all plots and selected as our study species (Fig. S4). For each common species in each plot (namely a population), 26-30 individuals (altogether 3672 individuals for all 130 populations; i.e., 26 species × 5 populations) outside the quadrats but within the plot and its 2 m extension were selected. The plant reproductive phenology was observed approximately weekly for altogether 25 times from the end of April to the middle of October (being transferred to Julian day in data analyses; individual reproductive phenology was observed in the typical, high-productivity and low-productivity plots on Julian day of 121, 129, 136, 143, 151, 158, 165, 171, 178, 184, 191, 197, 204, 210, 216, 222, 229, 236, 242, 249, 256, 264, 271, 278 and 286, and in the high- and low-diversity plots on Julian day of 122, 130, 137, 144, 152, 159, 166, 172, 179, 185, 192, 198, 205, 211, 217, 223, 230, 237, 243, 250, 257, 265, 272, 279 and 287). In each census, plant species or individuals would not be observed until they approached the peak flowering or fruiting time. The peak of an individuals’ flowering or fruiting was defined as the date (Julian day) when it reached its maximal number or proportion of open flowers and ripe fruits, respectively (Cara Donnaet al., 2014; Wolf et al., 2017). Specifically, for a plant individual, if nearly or more than half of its flowers were open in a certain census, Julian day of this census was defined as its peak flowering time and it was excluded from subsequent census until its peak fruiting time (nearly or more than half of its fruits were ripe). The population-level peak flowering or fruiting time was calculated from the individual mean. Because parts of individuals failed to flower or fruit, 2993 peak flowering and 2667 peak fruiting individuals were finally recorded and used in data analyses (data of individual flowering and fruiting time were seen in Supplementary Data 2 and 3), respectively.


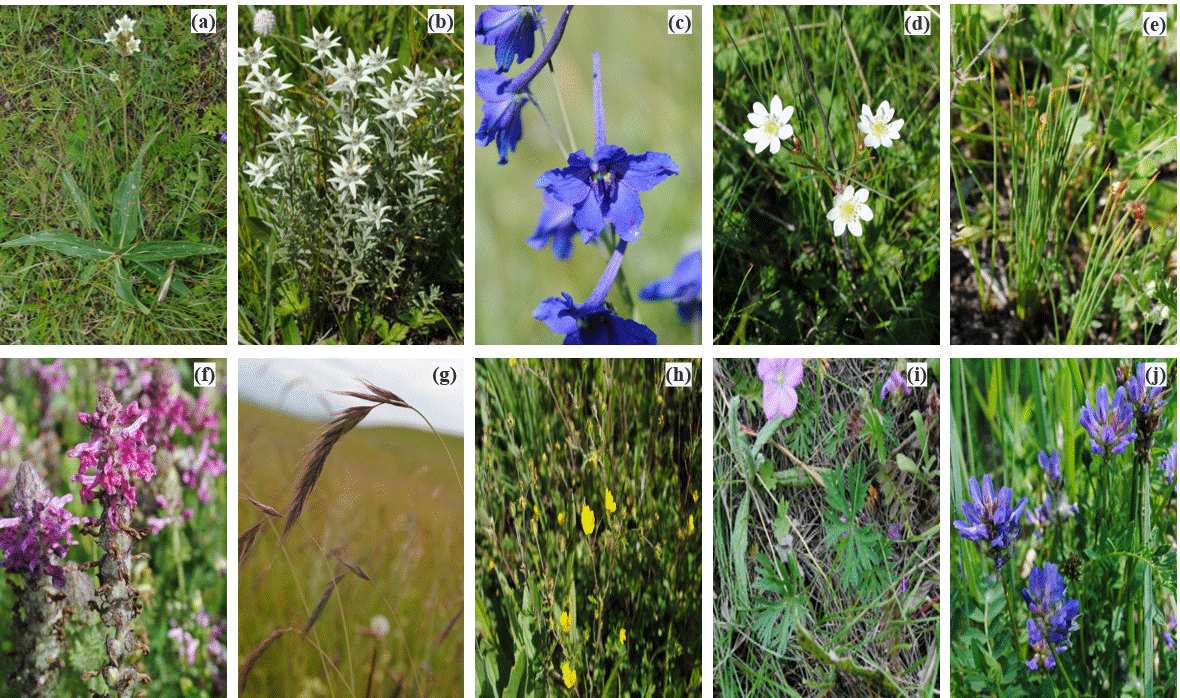


Fig. S4. Photographs of the main studied species. (a) *Gentiana straminea* Maxim.; (b) *Leontopodium haplophylloides* Hand.-Mazz.; (c) *Delphinium kamaonense* Huth; (d) *Anemone rivularis* Buch.-Ham. var. *flore-minore* Maxim.; (e) *Scirpus distigmaticus* (Kükenth.) Tang et Wang; (f) *Pedicularis kansuensis* Maxim.; (g) *Elymus nutans* Griseb.; (h) *Potentilla multifida* L.; (i) *Geranium pylzowianum* Maxim.; (j) *Astragalus adsurgens* Pall.

**Plant reproductive biomass allocation measurement**

For each population, 16-23 individuals (altogether 2518 individuals of all 130 populations) at fruit ripening stage (but no seed being scattered) were randomly selected outside the quadrats but within the plot and its 2 m extension to measure biomass allocation. In the field, each individual was harvested by cutting the stem at the soil surface and then divided into three parts: fruits, leaves and others (main stem). Each part of plant samples was weighed after drying for 48 hours at 60 °C. The reproductive biomass allocation of each individual was calculated by dividing its reproductive part biomass by its total biomass.

**SI References**

1. CaraDonna, P. J., Iler, A. M., & Inouye, D. W. (2014). Shifts in flowering phenology reshape an alpine plant community. Proceedings of the National Academy of Sciences, 111(13), 4916-4921.

2. Wolf, A. A., Zavaleta, E. S., & Selmants, P. C. (2017). Flowering phenology shifts in response to biodiversity loss. Proceedings of the National Academy of Sciences, 114(13), 3463-3468.

**Fig. S5.** The change in peak fruiting time of 26 grassland species in the high-productivity (□, square), low-productivity (○, circular), high-diversity (◇, rhombus) or low-diversity (△, triangle) community relative to the typical community. A positive (or negative) value for the change indicates that the variation in community productivity or diversity delays (or advances) fruiting time. Significant, marginally significant, and non-significant changes are represented in blue (*P* ≤ 0.05), orange (0.05 < *P* ≤ 0.1), and white (*P* > 0.10), respectively. Species are presented in order of their mean individual flowering time (Julian day) in the typical community. The numbers on the left side of figure represent the mean individual fruiting time of each species in the typical community.


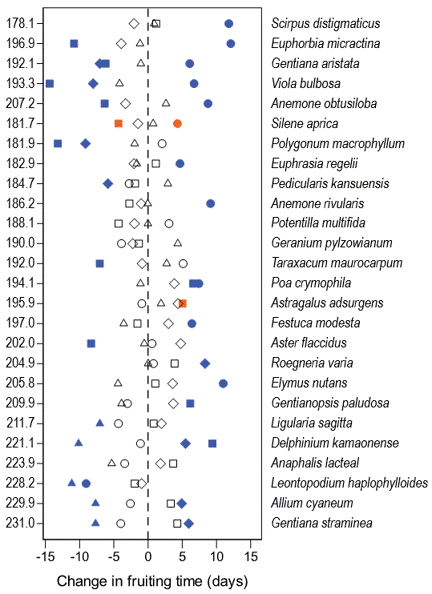


**Fig. S6.** The change in peak fruiting time for species with different biological attributes (A, early or late flowering; B, small or large individual size; C, anemophily or entomophily; D, clone or non-clone) in the high-productivity (HP), low-productivity (LP), high-diversity (HD) or low-diversity (LD) community relative to the typical community. Error bars indicate the 95% confidence intervals (CIs). Different letters show significant difference between species with different biological attributes in their fruiting time change with community productivity or diversity


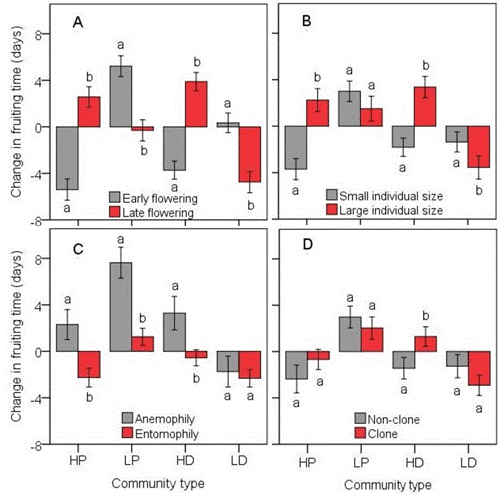

Supplement: pgae297_Supplementary_Data [file pgae297_supplementary_data.zip › PNASNEXUS-PNASNEXUS-2024-00082R-s01.docx]
